# Hypothalamic-Pituitary-Adrenal Axis Modulation of Glucocorticoids in the Cardiovascular System

**DOI:** 10.3390/ijms18102150

**Published:** 2017-10-16

**Authors:** Natalie G. Burford, Natalia A. Webster, Diana Cruz-Topete

**Affiliations:** Department of Molecular and Cellular Physiology, LSU Health Sciences Center, Shreveport, LA 71130-3932, USA; nburford3989@gmail.com (N.G.B.); nwebst@lsuhsc.edu (N.A.W.)

**Keywords:** hypothalamic-pituitary-adrenal axis, glucocorticoids, glucocorticoid receptor, mineralocorticoid receptor, smooth muscle cells, vascular endothelial cells, cardiomyocytes, heart

## Abstract

The collective of endocrine organs acting in homeostatic regulation—known as the hypothalamic-pituitary-adrenal (HPA) axis—comprises an integration of the central nervous system as well as peripheral tissues. These organs respond to imminent or perceived threats that elicit a stress response, primarily culminating in the release of glucocorticoids into the systemic circulation by the adrenal glands. Although the secretion of glucocorticoids serves to protect and maintain homeostasis in the typical operation at baseline levels, inadequate regulation can lead to physiologic and psychologic pathologies. The cardiovascular system is especially susceptible to prolonged dysregulation of the HPA axis and glucocorticoid production. There is debate about whether cardiovascular health risks arise from the direct detrimental effects of stress axis activation or whether pathologies develop secondary to the accompanying metabolic strain of excess glucocorticoids. In this review, we will explore the emerging research that indicates stress does have direct effects on the cardiovascular system via the HPA axis activation, with emphasis on the latest research on the impact of glucocorticoids signaling in the vasculature and the heart.

## 1. Activation of the Hypothalamic-Pituitary-Adrenal (HPA) Axis

**Figure 1:**
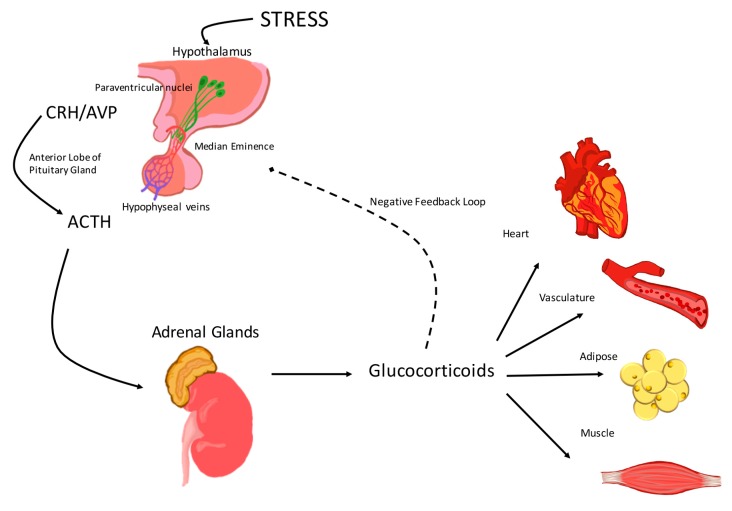
The activation of the hypothalamic-pituitary-adrenal (HPA) axis. Response to a stress stimulus is routed to the hypophysiotropic neurons in the paraventricular nucleus (PVN) of the hypothalamus. These neurosecretory cells release corticotropin-releasing hormone (CRH) and arginine vasopressin (AVP), which travel through the median eminence and hypophyseal portal vessels. Once CRH reaches the anterior pituitary, it binds CRF type 1 receptors of pituitary corticotroph cells. Adrenocorticotropic hormone (ACTH) is released into circulation, binds its receptors in the zona fasiculata of the adrenal cortex, and causes release of glucocorticoids. Glucocorticoids in circulation act on target cardiovascular tissues (including, the heart, the vasculature, adipose tissue and muscle) then feedback to the level of the central nervous system (CNS) to inhibit activation of the HPA axis.

Exposure to environmental, physical, or physiological stressors leads to the activation of the stress response, which comprises a complex interaction between components of the central nervous system (CNS) and peripheral systems, including the endocrine, immune, and cardiovascular systems [1]. In normal physiology, three structures modulate the response to stress: the paraventricular nucleus (PVN) of the hypothalamus, the anterior pituitary gland, and the cortex of the adrenal gland (Figure 1). Together these structures are referred to as the hypothalamic-pituitary-adrenal (HPA) axis [2]. The HPA axis plays a central role in the regulation of adrenal hormones that help to preserve or restore homeostasis.

Activation of the HPA axis initiates when the hypophysiotropic neurons localized in the PVN of the hypothalamus are stimulated to synthesize and secrete corticotropin-releasing hormone (CRH) [3] and arginine vasopressin (AVP) [4] (Figure 1). CRH and AVP are then released into the median eminence and transported to the anterior pituitary gland by the hypophyseal portal vessels (Figure 1). Once in the anterior pituitary gland, CRH binds corticotropin receptor factor (CRF) type 1 receptors (CRFR1), located on the pituitary corticotrophs. CRH binding to CRFR1 leads to the synthesis of adrenocorticotropic hormone (ACTH) from pre-pro-opiomelanocortin (pre-POMC) and induces ACTH secretion into the systemic circulation (Figure 1). AVP stimulates synthesis and secretion of ACTH by a variety of mechanisms that are discussed in detail later in this review. After release, ACTH binds to the melanocortin type 2 receptors (MC2-R) in the zona fasciculata of the adrenal cortex, triggering the synthesis and release of the primary stress hormones, glucocorticoids (cortisol in humans and corticosterone in rodents) (Figure 1).

An increase in glucocorticoid levels in response to internal and external stressors is advantageous in enabling the body to restore homeostasis. Glucocorticoids exert beneficial actions on the immune system, secondary metabolism, and cardiovascular function in response to stress. However, if glucocorticoid levels remain chronically elevated due to exposure to chronic stress, pharmacological treatments, or endocrine disorders, pathologies, including an increased incidence of cardiovascular disease, will develop [5]. Therefore, activation of the HPA axis and glucocorticoid secretion are tightly regulated by a negative feedback loop at the level of the hypothalamus and pituitary gland. The latter is the central mechanism whereby glucocorticoids inhibit ACTH synthesis and release, which in turn suppresses glucocorticoid secretion by the adrenal glands (Figure 1).

This review focuses on the effects of the hormones produced by the HPA axis (CRH, ACTH, AVP, and glucocorticoids) in the context of the cardiovascular system, with particular emphasis on the latest research on glucocorticoid effects in the vasculature and the heart.

## 2. The Hypothalamic-Pituitary-Adrenal (HPA) Axis and the Cardiovascular System

### 2.1. Corticotropin-Releasing Hormone (CRH)

The mammalian CRH is a 41-amino acid peptide produced and secreted by hypophysiotropic neurons of the PVN in the hypothalamus [3]. CRH is part of a family of structurally related peptides, known as corticotropin-releasing factors—among the members of this family are urocortins I, II, and III [6,7,8]. CRH is the primary regulator of HPA axis activation, as its secretion triggers the rapid release of ACTH and consequently the release of glucocorticoids by the adrenal gland, although AVP and catecholamines can additionally trigger ACTH secretion [4,9,10].

CRH signals through two distinct G-coupled receptors, CRFR1 and 2. These receptors display different pharmacological properties and expression patterns [11]. CRFR1 is highly expressed in the CNS, with higher levels of expression in the anterior pituitary gland and several areas of the brain [11,12]. In contrast, CRFR2 is expressed prominently in the periphery with limited expression in the CNS [13]. Based on these expression patterns and studies on CRFR1 knock out mice [14,15] CRFR1 is critical for induction of ACTH release from the anterior pituitary gland, while the role of CRFR2 in the regulation of the stress response is still unclear.

Concerning the systemic physiological role of CRH, studies on CRFR1, CRFR2, and CRH deficient mice show that CRH has effects on the immune, gastrointestinal, reproductive and cardiovascular systems [16]. Regarding CRH actions on the cardiovascular system, most CRH effects are indirect via glucocorticoid signaling. However, studies suggest that CRH has direct effects on the cardiovascular system. For example, CRH administration leads to increases in heart rate, cardiac output, and mean arterial pressure by stimulating norepinephrine (NE) and epinephrine (E) secretion [17,18]. There is also growing evidence that CRH may exert direct actions in the regulation of nitric oxide-dependent vasodilation and vascular permeability [19]. CRH can also signal through CRFR2 in cardiomyocytes and stimulate the production of atrial natriuretic peptide and brain natriuretic peptide during cardiac hypertrophy [19,20,21]. Nevertheless, most of the direct actions of CRH in the vasculature and the heart remain inconclusive and warrant further investigation. The role of the CRH paralogs, urocortins I, II, and III in the regulation of the stress response and their physiological effects are of particular interest. Also, further research is needed to fully understand the differences in down-stream signaling triggered by CRH binding to CRFR1 and CRFR2, perhaps the use of tissue-specific models will aid in dissecting the direct effects of CRH in the cardiovascular system.

### 2.2. Arginine Vasopressin (AVP)

AVP is a peptide hormone synthesized by the magnocellular neurons of the hypothalamus [22], also known as antidiuretic hormone. AVP is a potent vasoconstrictor that has a significant role in regulating water reabsorption and electrolyte balance [23]. Based on these effects of AVP, it is not surprising that this hormone has essential ramifications in the regulation of blood pressure [24]. AVP secretion is stimulated by “emergency situations,” including hemorrhage and other physiological conditions that induce profound hypotension [22,25,26]. In a state of shock, AVP induces rapid vasoconstriction of smooth muscle cells, which helps to maintain or increase blood pressure. However, AVP is also reported to work as a potent vasodilator of the endothelium of the coronary and pulmonary arteries [27,28], and the afferent arterioles of the kidney [29].

AVP exerts its vascular tone actions by signaling via tissue-specific G-protein coupled receptors. Based on their location, these receptors are classified as vasopressin receptor 1A (V1AR) (liver, kidney, peripheral vasculature, and brain), vasopressin receptor 1B (V1BR) also known as vasopressin receptor 3 (VPR3) (pituitary gland and brain), and the vasopressin receptor 2 (V2R), which is highly expressed in the kidney tubules.

Regarding AVP actions on the vasculature, studies show that AVP acts mainly as a vasoconstrictor. AVP binds to V1Rs on smooth muscle cells which leads to an increase in the expression of ATP-sensitive K^+^ channels (KATP); a decrease in the synthesis of nitric oxide (NO); and an increase in adrenergic and other vasoconstrictor agents, helping to restore vascular tone [26]. In contrast, AVP actions in the heart are more complex because AVP can cause coronary vasoconstriction or vasodilation [26]. In vitro and ex vivo studies show controversial results regarding the effects of AVP in the coronary vascular endothelium. Some studies report that AVP acts as a vasodilator [26,30,31], whereas other studies suggest that AVP acts as a vasoconstrictor [26,32,33]. More recent in vivo studies on the role of AVP provide clear evidence that AVP bolus injection leads to coronary vasodilation via AVP action on the endothelium tone [22,26]. On the other hand, studies on the effects of AVP in normoxia and hypoxia suggest that in a normoxic state AVP acts as a vasoconstrictor and significantly reduces coronary flow; in contrast, AVP leads to vasodilation during hypoxia, and therefore preserves coronary blood flow and improves myocardial function [26,34].

Together these results suggest that the vasoconstrictor/vasodilator effects of AVP are dependent on several factors, including the nature or location of the vascular beds, the type of receptor present in the target tissue, and the stress condition (e.g., normoxia/hypoxia). To dissect the actions of AVP in health and disease further research is needed.

In addition to the direct effects of AVP on the vasculature and the heart, AVP acts in conjunction with CRH to modulate the release of glucocorticoids. In response to stress, AVP is co-released from CRH nerve terminals at the median eminence and works in conjunction with CRH to enhance the release of ACTH [35,36,37]. AVP stimulates ACTH secretion by several mechanisms. The first mechanism involves AVP binding to V1BR, which leads to protein kinase C-mediated activation of CRH-stimulated cAMP production [37,38,39], and thereby secretion of pro-opiomelanocortin (POMC), the precursor of ACTH [40,41]. A second mechanism requires AVP up-regulation of CRH receptor gene expression in the pituitary gland, which increases CRH receptor synthesis [42,43]. Finally, a third mechanism is mediated by activation of GR receptor downstream signaling. This activation stimulates AVP-induced inositol phosphate formation, which in turn aids in maintaining corticotroph responsiveness under stress conditions (high cortisol levels) [36]. Importantly, although AVP actions on ACTH secretion are in general stimulatory, studies also show that AVP can inhibit CRH-stimulated *POMC* gene expression in corticotroph and melanotroph cells [37]. The mechanisms underlying these effects are unknown and require additional research.

### 2.3. Adrenocorticotropic Hormone (ACTH)

ACTH is a hormone derived from a 266-amino acid precursor, pro-opiomelanocortin (POMC) [44]. Depending on the availability (tissue distribution) of the cleavage enzymes, POMC yields ACTH, as well as other polypeptide hormones with distinct physiological activities, including β or γ-lipotropins, B-endorphins, α-melanocyte stimulating hormone (MSH) and corticotropin-like intermediate peptide (CLIP) [45]. Under normal physiology, the anterior pituitary releases ACTH in regular pulses of variable amplitude over a period of 24 h. In a similar fashion to cortisol, ACTH levels vary in an endogenous circadian rhythm, reaching a peak in the morning and declining throughout the day [46]. The synchrony between ACTH and cortisol secretion is maintained by glucocorticoids signaling back to the anterior pituitary to inhibit further ACTH secretion, and therefore prevents a chronic rise in glucocorticoid levels.

The pulsatile secretion of ACTH modulates glucocorticoid secretion by the regulation of gene transcription of the rate-limiting enzymes necessary for steroidogenesis, including steroidogenic acute regulatory protein (StAR) and cytochrome P450 side-chain cleavage (P450scc) as well as the MC2R and the MC2R accessory protein (MRAP) [47]. Therefore, ACTH pulsatile secretion plays a primary role in the gene regulation of critical enzymes and receptors involved in cortisol synthesis and secretion by the adrenal gland. Future studies on the effects of ACTH pulsatile secretion on the regulation of glucocorticoid receptor (GR) gene expression in peripheral tissues as well as on the expression of GR-target genes will provide additional insights into the physiological relevance of ACTH patterns of secretion.

The primary role of ACTH is regulating the synthesis and release of glucocorticoids by the adrenal gland [48]. Upon binding MC2R on the zona fasciculata of the adrenal cortex, ACTH induces the biosynthesis of glucocorticoids from cholesterol by the action of mitochondrial and smooth endoplasmic reticulum enzymes [49]. Therefore, excess ACTH production due to a pituitary corticotroph adenoma, an extrapituitary tumor (ectopic ACTH syndrome), or deficiency in ACTH production due to pituitary trauma, results in imbalances in glucocorticoid production, which have profound effects on cardiovascular health.

During a time of excessive production of ACTH, the adrenal gland will be overstimulated for the production of cortisol, resulting in hypercortisolism or endogenous Cushing’s syndrome [50]. Endogenous Cushing’s syndrome results in a vast array of cardiovascular complications arising from central obesity, insulin resistance, dyslipidemia, and a pro-coagulant state. Hypertension is the most common cardiovascular problem present in patients with endogenous Cushing’s syndrome. Prolonged hypertension leads to vascular damage (endothelial dysfunction), increased risk of premature atherosclerosis, coronary artery disease, cardiomyopathy and stroke [51,52]. Conversely, deficiency in ACTH production results in secondary adrenal insufficiency, which is characterized by a decrease in cortisol production. Low cortisol production may lead to acute cardiovascular collapse resulting from hypotension [50]. Based on these data, ACTH effects on the cardiovascular system are mainly mediated by glucocorticoid systemic effects; however, direct effects of ACTH via ACTH receptors are reported in human aortic endothelial cells [53]. These findings suggest that ACTH could affect blood pressure by modulating the vascular tone not only by regulating cortisol production but also by signaling directly through its receptors independent of glucocorticoids. In addition, studies on MC2R knockout mice show that ACTH exerts essential effects on adipose tissue metabolism [54,55]. Also, ACTH appears to act on the development and maintenance of the adrenal vasculature and regulates the adrenal blood flow by inducing the production of angiogenic and vasoactive agents from adrenocortical cells and adrenal mast cells. Therefore, ACTH may have a potential role in influencing systemic blood flow by binding to MC2R in the vascular endothelium. Future studies should aim to elucidate the systemic actions of ACTH in the vasculature.

### 2.4. Glucocorticoids

Activation of the HPA axis culminates with the release of glucocorticoids by the adrenal cortex. Once in the systemic circulation, glucocorticoids exert their physiological actions by binding the glucocorticoid receptor (GR, *NR3C1*), however in some scenarios glucocorticoids can also bind the closely related mineralocorticoid receptor (MR, *NR3C2*).

Most cell types and tissues throughout the body express GRs. In contrast, only selective tissues such as the epithelial cells of the kidney, colon, salivary and sweat glands, vascular endothelium, and cardiomyocytes express mineralocorticoid receptors (MRs) [56]. Aldosterone is the primary ligand for MR; however, aldosterone concentrations are in the range of 100- to 1000-fold lower than glucocorticoids [57]. Therefore, MR binding to aldosterone relies on the expression of the enzyme 11β-hydroxysteroid dehydrogenase type 2 (11β-HSD2) [58,59]. 11β-HSD2 converts cortisol to its inactive metabolite cortisone, which allows MR binding of aldosterone in tissues such as the kidney. While the vascular endothelium expresses 11β-HSD2, its expression in cardiomyocytes is almost undetectable; thus, the direct effects of glucocorticoids in the heart can be amplified, as they can signal through GR or MR [59].

Upon signaling activation in response to stress, glucocorticoids modulate the expression of numerous genes involved in metabolism, immune function, inflammation, growth, cognition, reproduction, and lung development, which allows the body to maintain homeostasis [5,57]. Glucocorticoids are well-known for their potent anti-inflammatory effects. Synthetic glucocorticoids are widely prescribed drugs for treating inflammatory disorders, such as asthma, allergy, sepsis, rheumatoid arthritis, ulcerative colitis, multiple sclerosis, and blood cancers [57]. Despite the extensive use of glucocorticoids in the clinic and their role as the central mediators of the stress response, their effects in the cardiovascular system are not fully characterized.

Elevation or deficiency in glucocorticoid levels results in metabolic and cardiovascular complications [56]. Increased glucocorticoid levels due to chronic stress, exogenous therapy, or endocrine disorders, commonly lead to obesity, metabolic syndrome, hypertension, and increase the risks for developing cardiomyopathies and subsequent heart failure [56,60,61]. Interestingly, glucocorticoid deficiency also leads to cardiovascular complications. For example, patients suffering from adrenal insufficiency (primary or secondary) can develop severe hypotension and suffer from a decrease in cardiac function that can lead to cardiac arrest [62]. In addition, recent genomic studies of patients carrying a polymorphism (A3669G) in exon 9 of the *NR3C1* gene (linked to decreased glucocorticoid signaling) show that this polymorphism increases the risk of coronary artery disease, pathological hypertrophy, and heart failure [63].

Data from animal and human studies suggest that glucocorticoids are essential to maintain cardiac contractile function. Adrenalectomy in rats and mice results in a decrease in cardiac contractile force. Administration of dexamethasone (a synthetic glucocorticoid lacking MR activity) [64,65] or corticosterone (primary glucocorticoid in rodents) [66] reverses this phenotype. Moreover, glucocorticoid administration enhances contractile tension and increases contraction and relaxation velocities in cardiac muscle [67]. Glucocorticoid therapeutic administration in pathological conditions, such as atherosclerosis, restenosis, chronic graft rejection, and ischemia appears to be beneficial as glucocorticoids inhibit the remodeling processes that lead to lesions by regulating the inflammation, angiogenesis, and cardiomyocyte apoptosis [68,69].

Together, these findings suggest that glucocorticoids certainly exert cardiovascular effects at the level of the vasculature and in cardiac tissue. Whether these effects are systemic or local, or if they are exerted via GR or MR, is still a topic of intensive investigation. In the next section, we will briefly review the latest research on glucocorticoid effects on the vasculature and the heart.

## 3. Glucocorticoid Effects on the Vasculature and the Heart

### 3.1. Glucocorticoid Effects on the Vascular Endothelium

The effects of glucocorticoids on the vasculature have been recognized for almost 40 years. Early studies in the 70’s and 80’s suggest that glucocorticoids exert essential effects on development, morphology, growth, proliferation, maintenance of vascular tone, and inflammation in smooth muscle and endothelial cells [70,71,72,73,74]. More recent studies show that the GR mediates most of these effects. GR directly modulates endothelial physiology by regulating the expression of adhesion molecules (VCAM-1, ICAM-1, and E-selectin), pro-inflammatory cytokine and chemokine production (IL-6, IL-17F, CXCL8 (IL-8), and CCL2 (MCP-1)), vasodilators (nitric oxide (NO)) and vasoconstrictors (angiotensin II or endothelin-1) that are involved in maintaining endothelial morphology and reactivity [75].

Studies demonstrate that glucocorticoids have essential effects on blood pressure regulation via the expression of GR in the vasculature. For example, glucocorticoids sensitize and potentiate the effects of catecholamines and other vasoactivators on vascular smooth muscle cells by suppressing the production of vasodilators, such as NO, through GR activation [76,77]. In vivo data also show that glucocorticoid administration leads to hypertension in mice by a mechanism involving GR inhibition of NO metabolites, NO_2_− and NO_3_− (indicators of total NO levels). These effects seem to be mediated by GR downregulating the gene expression of NO synthase III in the aorta, liver, and kidney [78]. Interestingly, endothelial-specific NO null mice do not develop hypertension in response to glucocorticoids, supporting the cross-talk between GR and NO system in the regulation of vasoconstriction [78].

The previous studies illustrate that glucocorticoids induce various effects on vascular smooth muscle both in vitro and in vivo. More recent studies by Goodwin et al., confirm these findings by utilizing a tissue-specific knockout of the GR in vascular smooth muscle. Studies of GR null mice show that intact GR signaling in vascular smooth muscle cells is important to control vascular tone in response to acute elevation of blood pressure [79]. Vascular smooth muscle GR specific knockout mice display a normal phenotype under basal conditions. Their growth, development, body weight, baseline heart rate (HR) and blood pressure (BP) are normal and similar to their littermate controls [79]. However, while acute glucocorticoid administration leads to an acute hypertensive response in control mice, the vascular smooth muscle GR specific knockout mice have an attenuated response to glucocorticoid-induced hypertension. These results suggest that vascular smooth muscle GR is critical in the pathogenesis of acute hypertension, but appears to play a less essential role in BP regulation under normal physiology. Additional studies, perhaps using a model of chronic glucocorticoid administration, are needed to further understand the effects of a long-term increase in glucocorticoid levels in BP regulation and to elucidate the molecular mechanisms underlying GR signaling actions in the vasculature.

Regarding the role of endothelial GR in blood pressure regulation [80] and systemic inflammation [81], studies by the same group show that GR deletion in endothelial cells results in increased expression of endothelial nitric oxide synthase (eNOS) and inducible nitric oxide synthase (iNOS). Elevated eNOs and iNOS increase NO production, which exacerbates hypotension in response to lipopolysaccharide (LPS) administration. This study demonstrates that endothelial GR is critical to regulate the magnitude of the inflammatory process in response to LPS, and that glucocorticoid anti-inflammatory effects on the endothelium are mediated via GR repression of NF-κB [81]. Thus, the actions of endogenous glucocorticoids acting via endothelial GR are essential for host protection from systemic inflammation or sepsis.

In addition to glucocorticoid effects on vasoconstriction/vasodilation and inflammation, studies on vascular endothelial cells reveal that glucocorticoids inhibit angiogenesis independently of its actions in regulating the inflammatory response by affecting cell proliferation, viability, and cell migration via GR regulation of the anti-angiogenic thrombospondin-1 [82]. Angiogenesis is a critical process for blood vessel growth and repair in ischemic cardiovascular diseases, therefore, understanding the effects of GR during this process is important to gain insight into the mechanisms underlying the effects of glucocorticoids on endothelial repair in ischemic disease. Finally, glucocorticoid actions on inflammatory cells (e.g., macrophages, leukocytes), including the regulation of cell death, and the expression of pro-inflammatory molecules, are also indirect mechanisms by which glucocorticoids influence endothelial cell response to injury [83].

**Figure 2:**
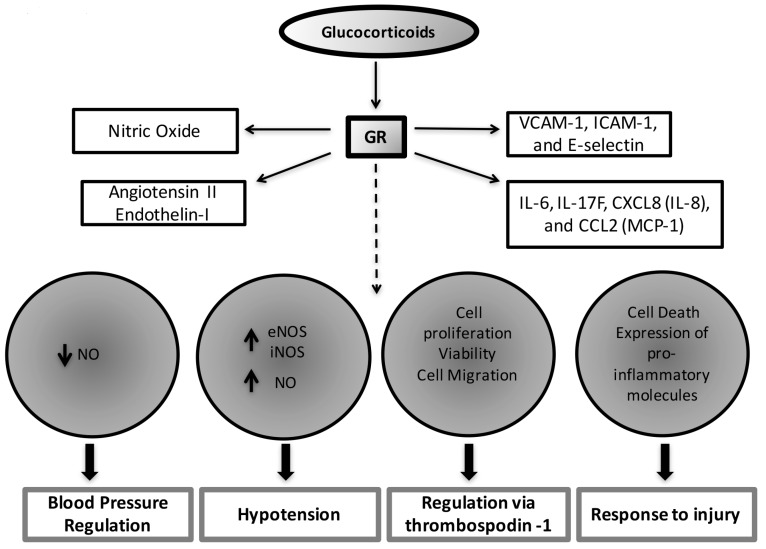
The direct and indirect effects of glucocorticoids on the vascular endothelium. glucocorticoids, through glucocorticoid receptors (GRs), regulate the expression of molecules critical to maintain the endothelium structural and functional properties. Among these molecules are adhesion molecules (VCAM-1, ICAM-1 and E selectin), pro-inflammatory cytokine and chemokine production (IL-6, IL-17F, CXCL8 (IL-8), and CCL2 (MCP-1)), vasodilators nitric oxide and vasoconstrictors angiotensin II and endothelin I [54,55,56,57,58,59]. Via the regulation of these mediators, GR exerts effects on blood pressure regulation, endothelial cell proliferation, viability, migration, cell death and the expression of inflammatory mediators in response to injury or infection. Dashed arrow: Effects of GR on the vasculature; Up arrow: up-regulation; Down Arrow: down-regulation.

The current experimental data indicate glucocorticoids have direct effects on the endothelium, and depending on the context these effects may be beneficial (e.g., infection) or detrimental (e.g., hypertension associated with chronically elevated levels of glucocorticoids). The known physiological roles of glucocorticoids on the vasculature are summarized in Figure 2.

### 3.2. Glucocorticoid Effects on the Heart

As previously discussed in this review, glucocorticoids undoubtedly exert both positive and negative effects on the cardiovascular system. However, the direct impact of these hormones on the heart is still ambiguous. Moreover, given that cardiomyocytes express very low or undetectable levels of 11β-HSD2 [58,59], it is unclear what effects are mediated via GR or MR.

Treatment of cardiomyocytes with glucocorticoids leads to both positive and negative effects. Beneficial effects of glucocorticoids are reported in the setting of hypoxia/reoxygenation [84]. Glucocorticoid treatment of cultured rat cardiomyocytes protects the cells against ischemia-induced apoptosis by inducing the expression of lipocalin-type prostaglandin D synthase (L-PGDS), which induces prostaglandin biosynthesis, limiting the inflammatory response and tissue damage [84]. In agreement with these results, studies by Ren et al., show that glucocorticoids can protect the cells from apoptosis in response to stress induced by starvation [85], but can also cause hypertrophy under certain conditions (in the presence of fetal bovine serum) [85]. Finally, the positive effects of glucocorticoids are recently highlighted in a study where glucocorticoids, via GR regulation of krüppel like factor (KLF)-13 [86], a zinc finger transcription factor involved in cardiac development, protect cardiomyocytes from DNA damage and cell death induced by cobalt(II) chloride hexahydrate (CoCl_2_·6H_2_O) and the antineoplastic drug doxorubicin [87]. Therefore, the effects of glucocorticoids on cardiomyocytes are dynamic and depending on the micro-environment and the nature of the stress stimulus their actions can turn beneficial or detrimental.

In terms of physiology, studies employing mouse models targeting cardiomyocyte GR or MR provide essential information regarding the in vivo role of glucocorticoid signaling in the heart. Elegant studies by Sainte-Marie Y et al. [88] using a tetracycline-inducible transgenic mouse model overexpressing hGR in cardiomyocytes demonstrate that although hGR-overexpression (3 times more than endogenous levels) does not lead to significant cardiac dysfunction or cardiac pathology (e.g., hypertrophy, fibrosis, or inflammation). The hGR-overexpressing mice display conduction defects characterized by electrocardiogram (ECG) abnormalities usually associated with bradycardia and atrioventricular block. Interestingly, this phenotype is reversed by shutting off hGR overexpression by treatment with doxycycline. Moreover, in vitro studies employing primary cardiomyocytes isolated from hGR-overexpressing mice reveal that the ECG abnormalities are the result of decreases in sodium and potassium currents and increases in L-type calcium currents, calcium transient amplitudes, and sarcoplasmic reticulum (SR) calcium content [88]. These results support a role for GR in the regulation of the heart cardiac conduction system. It is well accepted that activation of MR in the heart promotes adverse remodeling characterized by inflammation and fibrosis. Therefore, the results obtained in these studies open the possibility that the deleterious effects of elevated glucocorticoid levels observed in humans may be mediated through signaling via cardiomyocyte MR [89]. Indeed, gene expression studies employing cardiomyocytes isolated from GR (null/null) and MR (null/null) mice show that glucocorticoids regulate different genes in cardiomyocytes depending on whether they are signaling via GR or MR [90]. Thus, it is possible that activation of GR in the heart is beneficial while MR activation is deleterious.

Recent data from long-term adrenalectomized (ADX) mice also show that GR and MR activation by corticosterone or aldosterone has selective effects on the heart. In this model, corticosterone signaling via GR seems to play a predominant role in the regulation of left ventricular function and the expression of genes involved in maintaining cardiac homeostasis. In contrast, aldosterone activation of MR appears to play a predominant effect in the modulation of cardiac electrical activity [66]. These results are in agreement with studies on mouse models overexpressing MR in cardiomyocytes that demonstrate MR signaling regulates L-type calcium channel activity in cardiomyocytes [91]. These findings also correlate with data showing that aldosterone, through MR, promotes the occurrence of widespread and long-lasting Ca^2+^ sparks due to an increase in the activity of cardiomyocyte ryanodine receptors (Ryr2) [92]. Also, MR activation in the heart leads to alterations in the gene expression of ion channels, including potassium and calcium channels [93].

A novel mouse model lacking GR in cardiomyocytes (the cardiomyocyte-specific GR knock out) has been recently developed by Cidlowski and colleagues [94]. The cardiomyocyte-specific GR knockout mouse provides new insight into the physiological role of GR in the heart. These mice are born in the expected Mendelian ratios and display a normal phenotype for the first three months of life; however, they die prematurely (median survival ~7 months) from pathological cardiac hypertrophy that progresses to dilated cardiomyopathy and heart failure [94]. Functional studies reveal that around 3–6 months of age the cardiomyocyte-specific GR knockout mice exhibit significantly compromised heart function characterized by left ventricular systolic dysfunction. Associated with this functional deficit, the hearts of the knockout mice are enlarged (hypertrophic) and present significant dysregulation in genes involved in cardiovascular disease, including genes critical for cardiac contractility (Ryr2; dystrophin, Dmd), repressing cardiac hypertrophy (Krüppel like factor 15, Klf15), promoting cardiomyocyte survival (prostaglandin D2 synthase, Ptgds) and inhibiting inflammation (lipocalin 2, Lcn2; tristetraprolin, Zfp36) [94]. The results obtained by Oakley RH et al., demonstrate for the first time that stress has direct effects on the heart and that intact glucocorticoid signaling in cardiomyocytes is critical in normal physiology [94]. Studies need to be performed to elucidate further if the deleterious phenotype displayed by these mice results from inhibition of glucocorticoid signaling through cardiomyocyte GR or from excessive glucocorticoid activation of cardiomyocyte MR due to the lack of GR. The last scenario will correlate with studies on mice overexpressing hMR in the heart [91]. Mice overexpressing hMR have increased mortality compared to their wild-type littermate controls. These mice develop severe ventricular arrhythmias resulting from alterations in potassium transient outward current and L-type calcium current [91].

Loss of function studies have also been performed to characterize the role of MR in the heart. Cardiomyocyte inactivation of MR does not seem to have an effect on cardiac morphology or function in normal physiology [95,96]; however, when mice are challenged by ligating the left coronary artery, lack of MR attenuates cardiac dysfunction, cardiomyocyte death, and adverse remodeling [95,96]. These data indicate that cardiomyocyte MR expression is dispensable in maintaining cardiac homeostasis, but its activation during diseased states is deleterious and contributes to the pathology of ischemic heart disease. Similar results were obtained by Lother et al. [97] when cardiomyocyte-specific MR knockout mice were challenged in a model of chronic pressure overload (transverse aortic constriction (TAC)). MR null-hearts are protected from left ventricular dilation and dysfunction as compared to control mice [97]. In this model, MR deletion protects mice by preventing cardiac hypertrophy, fibrosis, apoptosis, and myocardial inflammation after TAC [97]. The role of MR has also been characterized in a model of hypertensive heart disease induced by deoxycorticosterone (DOC)/salt [98]. This study shows that the lack of cardiomyocyte MR prevents fibrosis and inflammation by inhibiting the expression of pro-inflammatory and fibrosis associated genes, and by decreasing inflammatory cell migration and infiltration to the myocardium [98].

**Figure 3:**
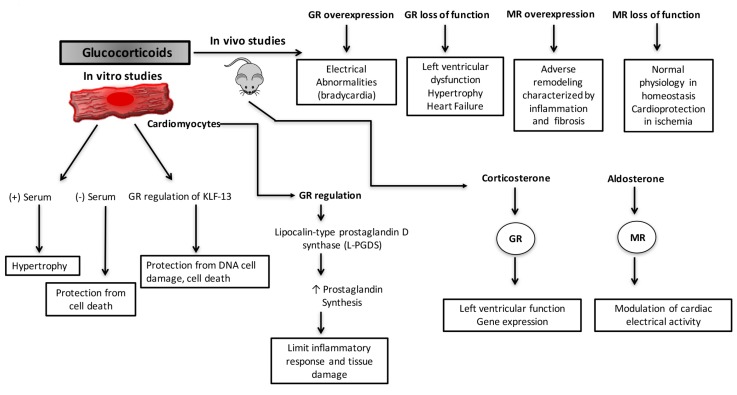
The physiological effects of glucocorticoids in the heart. Glucocorticoid treatment of cardiomyocytes has both positive and negative effects (right panel). In the setting of ischemia/reperfusion glucocorticoid treatment protects the cells against apoptosis by regulating the expression of lipocalin-type prostaglandin D synthase (L-PGDS), which induces prostaglandin biosynthesis, limiting the inflammatory response and tissue damage [68]. Also, glucocorticoids protect cardiomyocytes from apoptosis in response to starvation ((−) serum); however, glucocorticoids also lead to hypertrophy if cardiomyocytes are cultured in complete media ((+) serum) [69]. GR can also regulate pro-survival genes, for example, krüppel like factor (KLF)-13 [70], a zinc finger transcription factor involved in cardiac development. GR via KLF-13 regulation protects cardiomyocytes from DNA damage and cell death induced by cobalt (II) chloride hexahydrate (CoCl_2_·6H_2_O) and the antineoplastic drug doxorubicin [70]. Data from in vivo studies have shown that glucocorticoids have direct and indirect effects in the heart (left panel). Studies on long-term adrenalectomized mice have demonstrated that glucocorticoid signaling through GR exerts important effects on left ventricular function and cardiac gene expression, while aldosterone signaling via mineralocorticoid receptors (MR) appears to play a predominant effect in the modulation of cardiac electrical activity [50]. Gain/loss of function studies employing transgenic mouse models have demonstrated that glucocorticoids via cardiomyocyte GR are critical for normal physiology and play a role in maintaining cardiac electrical properties [72,78]. The role of MR in the heart has been demonstrated, employing a similar approach. MR activation under pathological conditions is detrimental and promotes adverse remodeling characterized by inflammation and fibrosis [75]. In contrast, loss of MR function leads to the attenuation of cardiac dysfunction, cardiomyocyte death and adverse remodeling in ischemia models [79,80,81,82].

The results obtained in these studies suggest that MR expression is deleterious under pathological conditions, but not under normal physiology. In contrast, expression of intact GR signaling in cardiomyocytes is critical for normal cardiac function and perhaps protects the heart from the detrimental effects of MR by preventing glucocorticoid over activation of MR signaling in the heart. Future studies are needed to further dissect the molecular and physiological effects of glucocorticoid signaling via GR or MR in the heart. The known physiological roles of glucocorticoids on the vasculature are summarized in Figure 3.

## 4. Concluding Remarks

The HPA axis mediates the physiological maintenance of homeostasis in stress conditions. This response is mediated by the activation of a complex regulatory system involving the endocrine, nervous and immune system. Dysregulation of these systems is linked to a vast array of pathologies including hypercortisolism, hypertension and subsequent vascular damage and cardiac arrest. In this review, we summarized the major hormones regulated by the HPA axis and their effects with a particular focus on the role of glucocorticoids in the vasculature and the heart. Characterizing the role of each of these systems and regulatory mechanisms will provide a better understanding of the body’s physiological response to stress, which may ultimately lead to new therapeutic targets.

CRH plays a central role in the regulation of the stress response via the stimulation of ACTH with effects on the gastrointestinal, reproductive and cardiovascular system. However, further studies need to confirm the direct role of action on the vasculature and the heart. Furthermore, while glucocorticoids are known to modulate the regulation of numerous genes involved in metabolism, immune function, inflammation, growth, cognition, reproduction, and lung development their effects on the cardiovascular system are not fully characterized. Additional studies are required to understand the molecular and physiological effects of glucocorticoid signaling via GR or MR in the heart.

Finally, one aspect lacking in each of the studies discussed in this review is the effect of the HPA axis and regulatory hormones on sexually dimorphic models. Further studies are necessary to determine whether hormone signal transduction pathways are altered and their subsequent physiological effects in sexually dimorphic scenarios.
